# An Integrated Metabolome and Transcriptome Analysis Reveal the Regulation Mechanisms of Flavonoid Biosynthesis in a Purple Tea Plant Cultivar

**DOI:** 10.3389/fpls.2022.880227

**Published:** 2022-05-19

**Authors:** SaSa Song, Yu Tao, LongHan Gao, HuiLing Liang, DeSong Tang, Jie Lin, YuChun Wang, Frederick G. Gmitter, ChunFang Li

**Affiliations:** ^1^College of Tea Science and Tea Culture, Zhejiang A&F University, Hangzhou, China; ^2^Institute of Food and Agricultural Sciences, Citrus Research and Education Center, University of Florida, Lake Alfred, FL, United States

**Keywords:** flavonoid, Zijuan, RNA-seq, metabolomics, UPLC-Q-TOF/MS, anthocyanins, quercetin, kaempferol

## Abstract

Purple tea plant cultivars, enrich with flavonoids and anthocyanins, are valuable materials for manufacturing tea with unique color and flavor. Researchers found that ‘Zijuan’ leaves changed from purple to green mainly caused by the decreased flavonoids and anthocyanins concentrations. The mechanism of flavonoids and anthocyanin biosynthesis has been studied in many purple tea plant cultivars and the key genes which regulated the biosynthesis of flavonoid and anthocyanins in different purple tea plant cultivars were quite different. Also, the molecular regulation mechanism underlying the flavonoids and anthocyanins biosynthesis during leaves development and color changes is less-thoroughly understood. In this study, an integrative analysis of transcriptome and metabolome was performed on the purple leaves and green leaves of ‘Zijuan’ tea plant to reveal the regulatory networks correlated to flavonoid biosynthesis and to identify key regulatory genes. Our results indicated that the ‘Zijuan’ new shoots leaves were purple might be due to the copigmentation of quercetin and kaempferol derivatives. In ‘Zijuan’ tea plant cultivar, flavonoids metabolites concentrations in purple leaves and green leaves were significantly influenced by the genes involved in flavonoid biosynthesis, transcriptional regulation, transport, and hormone response. Transcription factors including NAC008, MYB23, and bHLH96 and transporters such as ABC transporter I might be responsible for the flavonoid and anthocyanins accumulation in purple leaves. This study provides a new insight into the metabolism and molecular mechanisms underlying flavonoid and anthocyanin biosynthesis in tea plant.

## Introduction

‘Zijuan’ [*Camellia sinensis* var. *asssamica* (Masters) kitamura] is a special broad-leaf variety of tea plant that produces new shoots that are purple-colored and mature leaves that are green-colored. A decreased concentration in the flavonoids and anthocyanins causes the leaf color change from purple to green (Wang et al., 2017). The purple tea plant leaves were found to have a significantly higher concentration of total phenolic compounds, flavonoids, and anthocyanins, whereas the green leaves were found to have a higher concentration of porphyrin, chlorophyll, and carotenoids (Kerio et al., 2013; Kilel et al., 2013; Shen et al., 2018). Anthocyanins not only play various human health-correlated biological functions, such as acting as antimicrobial agents and antioxidants, lowering blood lipids, and preventing colorectal cancer, but also protect plants against various biotic and abiotic stresses, including low phosphate stresses and cold/freezing (Hsu et al., 2012). Therefore, the high concentration of flavonoids and anthocyanins has been employed as one of the tea plant breeding objects.

The combination analysis of high-throughput functional genomics large-scale datasets has been used on the analysis the functions of genes which might involve in plant metabolism regulating (Zhang et al., 2018). The integration of transcriptome and metabolome datasets by correlation and clustering analyses has been shown to be a useful approach to connect genes with metabolites in many plants, including crops (Kovinich et al., 2011), trees (Hamanishi et al., 2015; Shen et al., 2018; Zhang et al., 2018), fruits (Savoi et al., 2016), and potatoes (Cho et al., 2016). In tea plants, using metabolites and transcriptional profiling analysis found that glycosyl might determine the stable accumulation of anthocyanins in purple tea plant cultivar and anthocyanidin synthase, anthocyanidin 3-*O*-glucoside 6″-*O*-acyltransferase, anthocyanidin 3-*O*-glucosyltransferase, as well as transcriptional factors such as WRKY, and MYB were take part in transforming anthocyanins and increasing anthocyanins concentrations (Mei et al., 2020). Anthocyanin is biosynthesized through the phenylpropanoid and flavonoid pathways. Phenylalanine ammonia lyase (PAL) deaminates phenylalanine into cinnamic acid and involved in the biosynthesis of plant phenolics. In tea plants, the PAL activity in the purple leaves was shown to be two times higher than that of the green leaves (Li et al., 2017). A R2R3-MYB transcription factor CsMYB6A were found could activate the expression of flavonoid biosynthesis pathway genes and resulted the increasing of anthocyanins concentrations in purple tea plant leaves (He et al., 2018). Although a great effort has been focused on the genes implicated in flavonoids biosynthesis, with several key genes being identified (Li et al., 2017; Wei et al., 2019), insights into the molecular regulation mechanism of flavonoid and anthocyanin biosynthesis and transportation remain to be less-thoroughly uncovered.

Researchers have found that the vital genes which involved in regulating the biosynthesis of anthocyanins in different purple tea plant cultivars were quite different. The expression levels of *4-coumarate CoA ligase* (4*CL*), *chalcone synthase* (*CHS*), *flavonoid 3′-monooxygenase* (*F3′H*), and *flavonol synthase* (*FLS*) were in accordance with the dynamic concentration of delphinidin and cyanidin in ‘Zijuan’ (Mei et al., 2020). In ‘ZiYan,’ the expression levels of 4*CL*, *chalcone isomerase* (*CHI*), *flavanone 3-hydroxylase* (*F3H*), *F3′H*, and *flavonoid 3′,5′-hydroxylase* (*F3′5′H*) were consistent with the dynamic concentration of delphinidin and cyanidin (Mei et al., 2020). This study focused on the representative purple tea plant cultivar ‘Zijuan.’ Combined analysis of the metabolome and transcriptome was performed in the purple and green leaves to discover the regulatory networks correlated to flavonoid and anthocyanin biosynthesis and to identify the pivotal regulatory genes. Transcriptional and metabolic changes were analyzed statistically using metabolite-gene correlated networks. Transcriptional profiles and metabolite profiles were obtained using high-throughput RNA-sequencing and liquid chromatography-tandem mass spectrometry (LC-MS/MS), respectively. Flavonoids and anthocyanins were identified and quantified using molecular formula-based masses and MS2 spectra. Correlation analysis of differential flavonoids concentrations and differentially expressed genes (DEGs) showed the strong correlations between flavonoid compounds and DEGs, which were involved in flavonoid biosynthesis, transcriptional regulation, transport and hormone response. The network of differential flavonoids and DEGs discovered a regulatory system in the purple leaves of ‘Zijuan,’ provides a novel insight into the molecular mechanisms underlying flavonoid and anthocyanin biosynthesis in tea plant.

## Materials and Methods

### Plant Materials

**FIGURE 1:**
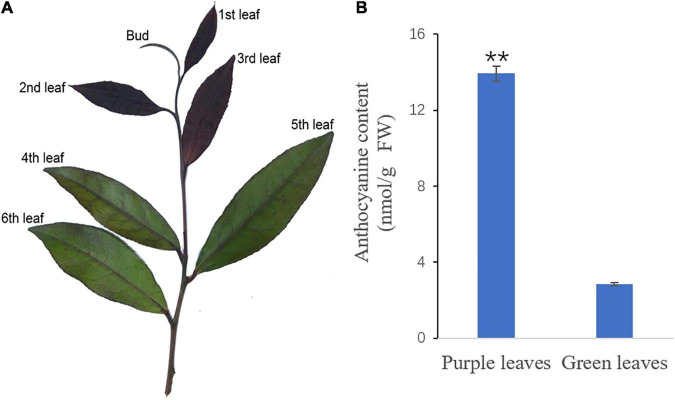
The new shoot of ‘Zijuan’ and the total anthocyanin concentrations in the purple and green tea plant leaves. **(A)** The purple leaves (2nd leaf and 3rd leaf) and the green leaves (4th leaf and 5th leaf) from ‘Zijuan’ tea plant new shoots were analyzed in this study. **(B)** The total anthocyanin concentrations in the purple and green leaves. **represents a significant difference between the purple leaves and the green leaves in the tea plant (*p* < 0.01).

‘Zijuan’ tea plants were grown on the tea plantation of the Zhejiang A&F University, Zhejiang province, China. The 2nd and 3rd leaves with a purple color and the 4th and 5th leaves with a green color were collected from at least ten tea plants on April 10, 2017 (Figure 1A). Six biological replicates were used for each chemical assay, and three biological replicates were applied for the transcriptome analysis. All the tea plant leaf samples were immediately frozen in liquid nitrogen and then stored at -80°C before analysis.

### Anthocyanin Analysis

The concentration of total anthocyanin in purple leaves and green leaves of ‘Zijuan’ were quantified using a spectrophotometer (UV-160 Shimadzu, Japan) (Proctor, 1974). With intermittent shaking (10 s on a vortex mixer), 10 ml of 0.1 M HCl in ethanol was used to extract 0.1 g of leaf sample at 60°C for 30 min. After that, the extract solution was filtered, and the absorbance was measured at 530, 620, and 650 nm, respectively. The concentration of anthocyanin was determined according to the following formula: ΔA = (A^530^ – A^620^) – 0.1(A^650^ – A^620^). The concentration of anthocyanin were measured by the following formula: total anthocyanin (μmol/g) = (ΔA × 100)/(4.62 × sample weight). The concentration of anthocyanin is presented as the mean ± standard deviation (SD) (*n* = 6). Significance was determined using Student’s *t*-test (*p* < 0.05) for differences between the purple leaves and the green leaves.

### Metabolomic Data Acquisition by UPLC-Q-TOF/MS

A total of 50 mg per leaf sample was added to 1 ml of 70% methanol with 20 μl of internal standard (0.03 mg ml^–1^L-2-Cl-phenylalanine), and then ground at 60 Hz for 2 min. Total metabolites in the leaves were extracted in an ultrasonic bath for 30 min and then centrifuged at 15,000 *g* for 10 min. The upper phase of the aqueous methanol extract was filtered through a 0.22 μm PTFE filter. A total of 3 μL of the extract was injected into an Agilent 1290 Infinity UHPLC. The metabolites were separated on a Waters ACQUITY UPLC BEH Amide Column (1.71 μm, 100 mm × 2.1 mm). The Mobile phase A was 25 mM ammonium acetate in water and 25 mM ammonium hydroxide, and mobile phase B was acetonitrile. The flow rate was 0.5 ml/min, and the gradient profile was 5% A from 0 to 0.5 min, 35% A from 0.5 to 7 min, 60% A from 7 to 8 min, 60% A from 8 to 9 min, and 5% A from 9.1 to 12 min. The metabolomic data were acquired in both electrospray ionization negative (ESI-) and positive (ESI+) modes. Ion spray voltage was –4.0 kV in ESI- and 5.0 kV in ESI+, the nebulizer gas was 60 psi, the heater gas was 60 psi, the curtain gas was 35 psi, and the turbo spray temperature was 650°C.

### Data Processing and Differential Metabolites Identification

UPLC-Q-TOF/MS raw data files were imported into XCMS^1^ to produce a peak table that included information on the retention time, MS intensity, and mass-to-charge ratio (m/z) for each metabolite. The intensities of mass peaks for each sample were normalized by inputting the datasets of UPLC-Q-TOF/MS into the SIMCA-P+ 14.0 software package (Umetrics, Umea, Sweden). Principal component analysis (PCA) was conducted to detect the intrinsic variation between the purple leaves and the green leaves. Orthogonal partial least squares discriminant analysis (OPLS-DA) was used to discriminate and characterize the purple and the green leaves. Differential metabolites were identified as those with a variable influence on projection (VIP) value greater than 1 and with a *p* value less than 0.05. Differential metabolites were annotated with METLIN.^2^ The identified differential metabolites were enriched into distinct metabolic pathways according to the Kyoto Encyclopedia of Genes and Genomes (KEGG) database to analyze the metabolic differences in purple leaves and green leaves in ‘Zijuan.’

### Transcriptome Library Construction and RNA-Sequencing

Total RNA was isolated from three purple leaf samples and three green leaf samples in ‘Zijuan’ tea plant, using RNeasy Plus Mini Kit (Qiagen, Valencia, CA, United States). The libraries were constructed, and paired-end sequencing was performed on the Illumina HiSeq™ 2000 platform, according to the manufacturer’s instructions. Fluorescent image analysis, base calling, and quality value calculations were performed using the Illumina Pipeline with default settings. HISAT2 first aligned the reads with the tea plant (*C.* s*inensis* var. s*inensis*) genome sequence, and then StringTie (v2.0.6) assembled the reads into unigenes (Daehwan et al., 2015; Mihaela et al., 2015; Wei et al., 2018; Xia et al., 2020). Unannotated transcriptional regions were identified by comparing with the original genome annotation (Wei et al., 2018; Xia et al., 2020) and were named as Camellia_sinensis.newGene.

### Unigene Functional Annotation and Expression Analysis

Using the BLASTp algorithm, all unigenes were annotated by searching against the Swiss-Prot protein sequence database (Swiss-Prot), and proteins with the highest sequence similarity to given unigenes were retrieved. In the same way as outlined above, the unigenes were blast with the Kyoto Encyclopedia of Genes and Genomes database (KEGG), and the unigenes that enriched with KEGG database were retained for detailed pathway analysis. The expression levels of each unigene were calculated based on fragments per kilobase of exon per million mapped reads (FPKM) values using StringTie (Mihaela et al., 2015). The significance of each gene expression level difference between the purple and the green leaves was assessed using the DEseq (Wang et al., 2010) on the FPKM values from three cDNA libraries from the purple leaves and three cDNA libraries from the green leaves. To identify the DEGs between the purple leaves and green leaves, a false discovery rate (FDR) ≤ 0.01 and Fold Change ≥ 4 was used to judge the gene expression differences significance between the purple leaves and green leaves. The DEGs annotated as involved in flavonoids pathway were clustered hierarchically according to their log FPKM values. Using KOBAS (Mao et al., 2005), the statistical enrichment of DEGs in KEGG pathways were tested.

### Quantitative Real-Time PCR Analysis

Total RNA of the purple leaves and green leaves in the ‘Zijuan’ tea plant was isolated using the RNeasy Plus Mini Kit (Qiagen). To remove DNA prior to cDNA synthesis, the samples were treated with TURBO DNase (Ambion, Austin, TX, United States). The quantitative Real-Time PCR (qRT-PCR) reaction mixture included 2 μL template cDNA, 5 μM each of the forward and reverse primers, and 10 μL SsoFast EvaGreen Supermix. PCR amplification was conducted at 60°C as annealing temperature using ABI 7500 real-time PCR system (Applied Biosystems). Each analyzed unigene was tested with three biological replicates and three technical replicates. The quantitative data were analyzed using the 2^–ΔΔCT^ method, with the GAPDH gene as an internal standard. The primer pairs used for the qRT-PCR are listed in Supplementary Table 5.

### Correlation Analysis of the Transcriptome and the Metabolome

To obtain key genes involved in flavonoid biosynthesis, DEGs and differentially accumulated flavonoids between the purple leaves and the green leaves were selected for integrative analysis. Pearson correlation coefficients (PCC) between differential metabolites and DEGs, and *p* value of PCC (PCCP) were calculated. The Pearson correlation coefficients (PCC) ≥ 0.90 or ≤-0.90 and the PCCP < 0.05 were selected. The coexpression network was exhibited using Cytoscape (version 2.8.2) (Shannon et al., 2003).

## Results

### Comparison of Total Anthocyanin Concentration Between Purple and Green Leaves in ‘Zijuan’ Tea Plant

The new shoots of ‘Zijuan’ were dark purple, but as they developed, they became completely green. The purple leaves (2nd and 3rd leaves), green leaves (4th and 5th leaves), and total anthocyanin concentration in these leaves are presented in Figure 1. The total anthocyanin content in the green leaves was 2.85 nmol/g, while that in the purple leaves was significantly greater, 13.93 nmol/g (Figure 1B).

### Metabolic Differences Between the Purple Leaves and the Green Leaves

**FIGURE 2:**
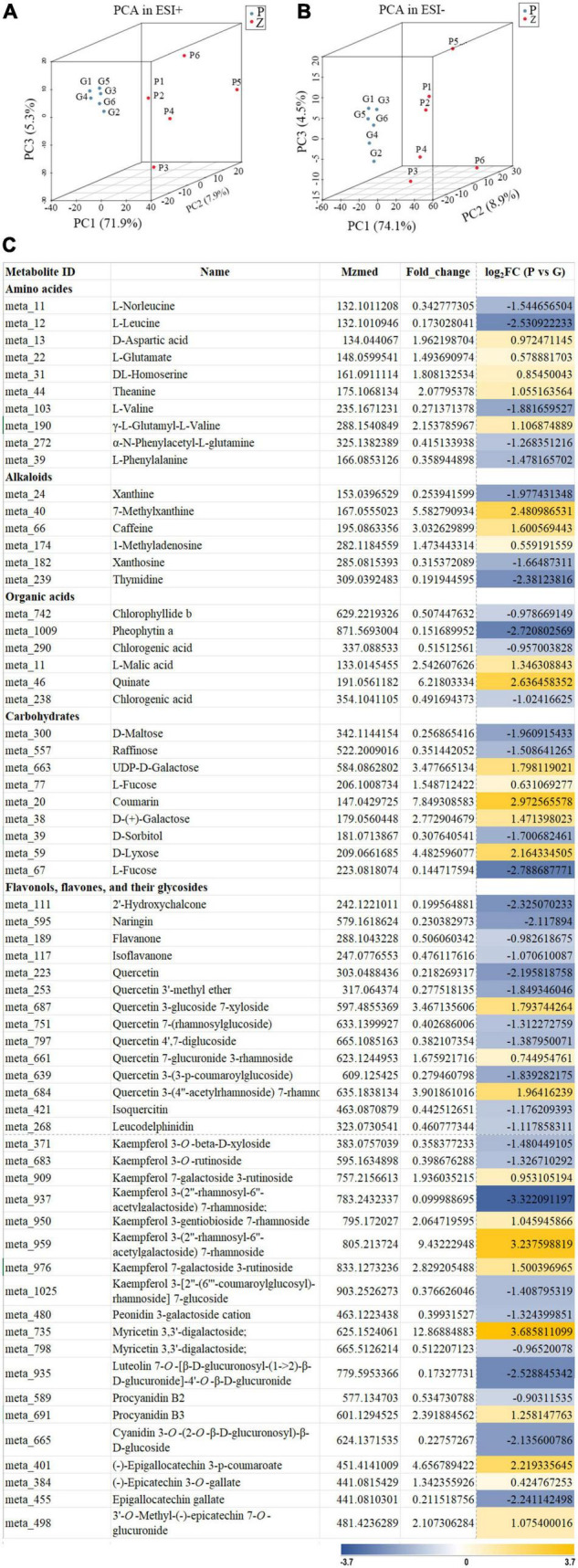
Principal component analysis score plot and differential metabolites of the purple leaves and the green leaves in ‘Zijuan’ tea plant. **(A)** PCA score plots derived from metabolite ions obtained from ESI+ modes. **(B)** PCA score plots derived from metabolite ions obtained from ESI- modes. P1–P6 represented six purple leave samples and G1–G6 represented six green leave samples. **(C)** Relative fold change of differential metabolites in the purple-leaf and green-leaf in ‘Zijuan’ according to UPLC-Q-TOF-MS analysis.

Principal component analysis was used to compare the metabolite composition differences between the purple leaves and the green leaves, based on the metabolic database got from UPLC-Q-TOF/MS in the ESI+ and ESI- mode. The purple and green leaves were obviously separated in the PC1 × PC2 × PC3 score plots (Figures 2A,B). The first principal components (PC1) in ESI+ mode (71.9% of the total variables) and in ESI- mode (74.1% of the total variables) was clearly separated between the purple and green leaves. Furthermore, OPLS-DA was used for modeling the differences between the purple leaves and the green leaves in ‘Zijuan.’ A total of 64 differential metabolites were identified, 27 of which were upregulated and 37 of which were downregulated when comparing the purple with the green leaves (Figure 2C).

**FIGURE 3:**
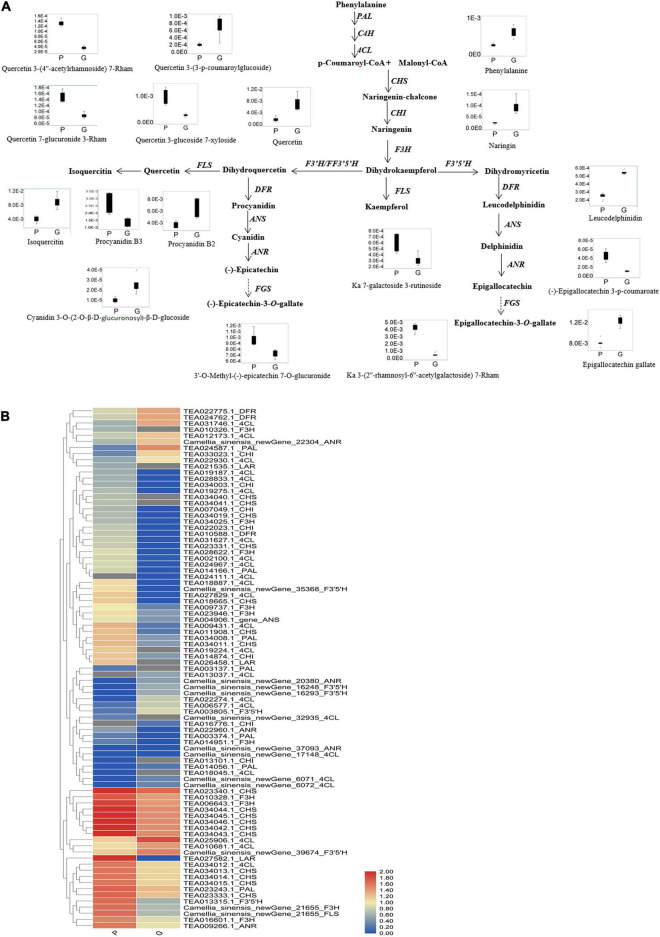
Flavonoid biosynthetic pathway and the expression level of DEGs. **(A)** Flavonoid and anthocyanin biosynthetic pathway. This pathway was constructed based on the KEGG pathway and literature references. Ka, kaempferol; Rham, rhamnose; Box-and-whisker plots are shown for the concentration changes in the phenylprophanoids, flavone glycosides, flavonol glycosides, dihydroflavonol glycosides, and anthocyanins in the purple leaves (P) and the green leaves (G). The maximum and minimum values of each metabolite concentration among the six biological replicates are represented at the upper and lower ends of the whisker, respectively. **(B)** Heat map of the expression levels of the flavonoid biosynthetic unigenes in the purple leaves and the green leaves. The annotations are displayed on the right side of each unigene. The scale represents the logarithms of the FPKM (fragments per kilobase of exon model per million reads mapped) values of each unigene. The unigenes were clustered by the Pearson correlation.

Based on the mass ion peaks detected in the UPLC-Q-TOF/MS analysis, the metabolites in the flavonoid biosynthesis pathway were selected. These selected flavonoids and anthocyanins, and correlated metabolites were arranged to their corresponding positions in the flavonoid biosynthesis pathway that was constructed according to KEGG and literature references (Figure 3). The concentrations of the metabolites in the flavonoids biosynthesis pathways were significantly different between the purple leaves and the green leaves. Procyanidin B3, kaempferol 7-galactoside 3-rutinoside, kaempferol 3-(2″-rhamnosyl-6″-acetylgalactoside) 7-rhamnoside, quercetin 7-glucuronide 3-Rham, quercetin 3-glucoside 7-xyloside, and quercetin 3-(4″-acetylrhamnoside) 7-rhamnoside were more abundant in the purple leaves, whereas phenylalanine, naringin, quercetin, quercetin 3-(3-p-coumaroylglucoside), isoquercitin, leucodelphinidin, procyanidin B2, cyanidin 3-*O*-(2-*O*-β-D-glucuronosyl)- β-D-glucoside, and epigallocatechin gallate were abundant in the green leaves (Figure 3A). 3′-*O*-methyl-(-)-epicatechin 7-*O*-glucuronide, (-)-epigallocatechin 3-p-coumaroate, and (-)-epicatechin-3-*O*-gallate were found at significantly higher concentrations in the purple leaves than in the green leaves (Figure 3A).

### Differential Expression Genes Between the Purple Leaves and the Green Leaves

**FIGURE 4:**
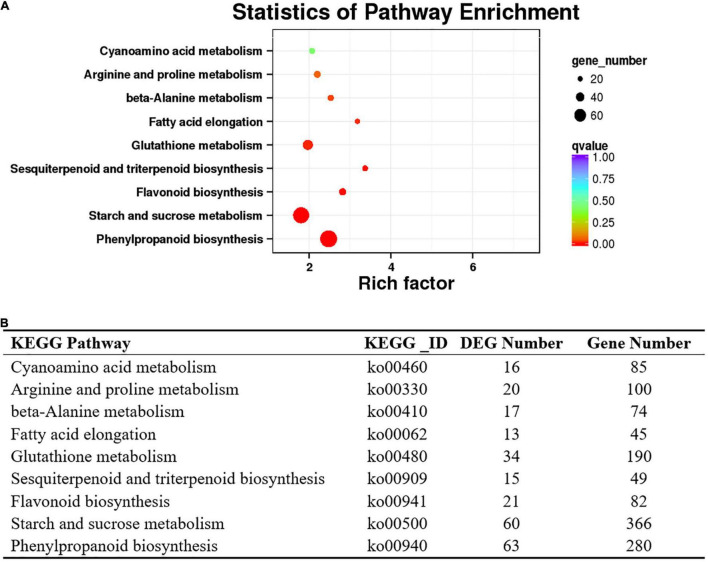
Kyoto Encyclopedia of Genes and Genomes database enrichment analysis of DEGs in the purple-leaf and green-leaf in ‘Zijuan’. **(A)** The KEGG enrichment analysis of the DEGs showed the pathways with the significant enrichment. **(B)** List of the DEGs number which were enriched in the KEGG pathways.

Transcriptome analysis showed that 4,729 transcripts were differentially expressed in the purple and green leaves. Compared with the green leaves, there were 1,671 upregulated and 3,058 downregulated transcripts in the purple leaves (Supplementary Table 1). To further determine the functions of the DEGs, KOBAS (Mao et al., 2005) was applied to analyze the statistical enrichment of DEGs in KEGG pathways, and the phenylpropanoid biosynthesis, starch and sucrose metabolism and flavonoid biosynthesis were the three most prominent pathways identified (Figures 4A,B). DEGs involved in flavonoid biosynthesis, such as *CHS*, *FLS*, *flavonoid 3′5′ hydroxylase* (*F3′5′H*), and *anthocyanidin reductase* (*ANR*), were enriched in the phenylpropanoid biosynthesis and flavonoid biosynthesis pathways (Supplementary Table 2).

**FIGURE 5:**
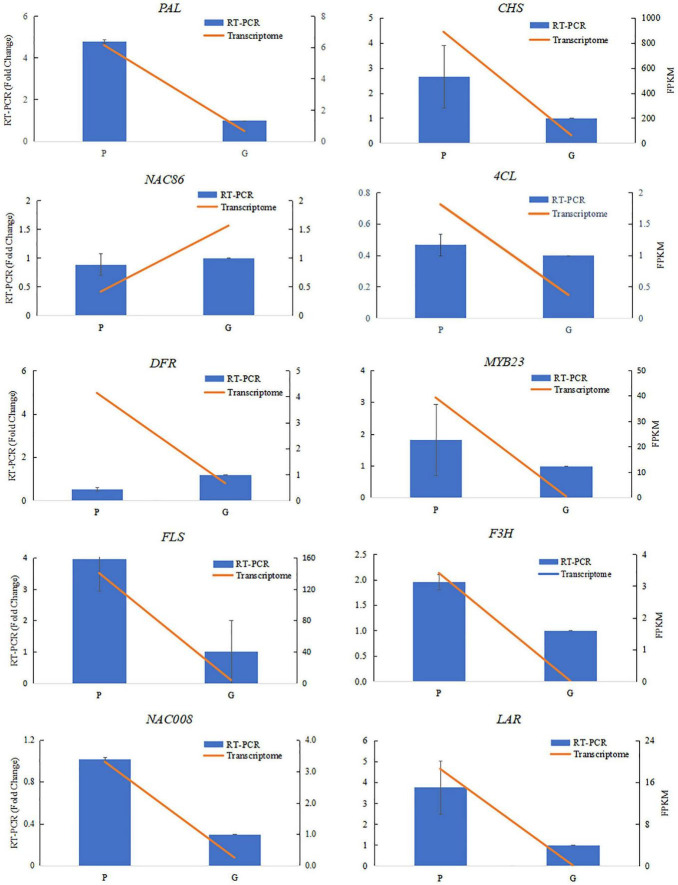
Verification of RNA-sequencing results using qRT-PCR assays. Ten differentially expressed genes were selected from the flavonoid biosynthesis pathway and transcriptional factors involved in flavonoid biosynthesis. The qRT-PCR data were normalized using the “housekeeping” gene GAPDH. The fold change in normalized GAPDH levels in the purple-leaf (P) and green-leaf (G) in ‘Zijuan’ was calculated. The qRT-PCR primers are listed in Supplementary Table 5.

Differentially expressed genes involved in flavonoid and phenylpropanoid pathways were clustered by the Pearson correlation based on the expressional level of each DEG in the purple or green leaves (Figure 3B and Supplementary Table 3). The expression level of *PAL* (TEA014166.1, TEA023243.1, TEA034008.1, TEA003137.1, TEA003374.1) and *4CL* (Camellia_sinensis_newGene_17148, Camellia_ sinensis_newGene_32935, TEA009431.1, TEA018887.1, TEA019187.1, TEA019224.1, TEA019275.1, TEA024967.1, TEA002100.1, TEA027829.1, TEA028833.1, TEA031627.1, TEA034012.1) in the purple leaves were significantly higher than in the green leaves (Figure 3B). The expression level of *CHI* (TEA007049.1, TEA013101.1, TEA014874.1, TEA022023.1_CHI, TEA034003.1) in the purple leaves were also significantly higher than in the purple leaves. All the DEGs encoding F3H (Camellia_sinensis_newGene_21655, TEA006643.1, TEA009737.1, TEA010326.1, TEA010328.1_F3H, TEA014951.1_F3H, TEA016601.1_F3H, TEA023946.1_F3H, TEA028622.1_F3H, TEA034025.1_F3H) were expressed at a higher level in the purple leaves than in the green leaves. The transcription levels of *FLS* (Camellia_sinensis_newGene_21655, TEA006643.1_gene, TEA010328.1_gene, TEA016601.1_gene) in the purple leaves were significantly higher than those in the green leaves. The differential gene expression levels between purple and green leaves were validated using quantitative real-time PCR (qRT-PCR) (Figure 5) with gene-specific primers (Supplementary Table 5). From the RNA isolated from the purple leaves and the green leaves, the genes involved in flavonoid biosynthesis and regulation, including *NAC008*, *PAL*, *4CL*, *F3H*, *leucoanthocyanidin reductase* (*LAR*), *FLS*, and *CHS* were found to be highly expressed in the purple leaves, whereas *NAC86* was highly expressed in the green leaves.

### Correlation Analysis Between Differentially Expressed Genes and Differential Metabolites in the Flavonoid Biosynthesis Pathway

**FIGURE 6:**
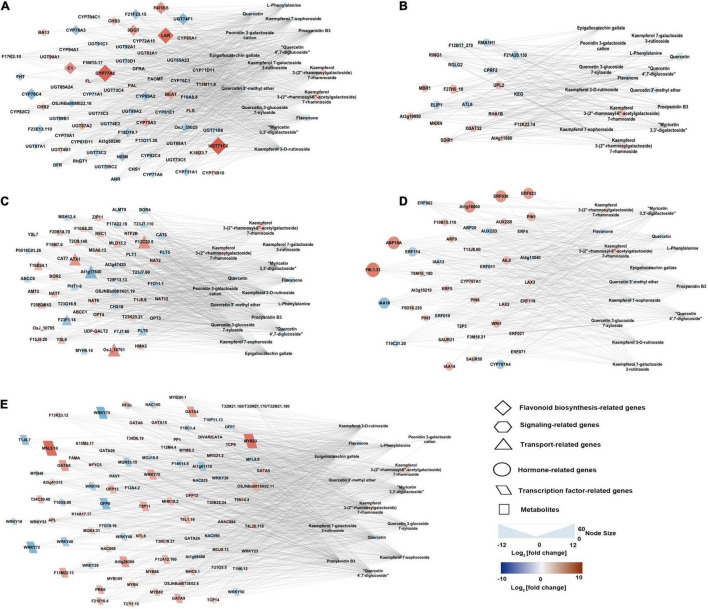
Connection network between genes (regulatory and structural) and flavonoid- and anthocyanin-correlated metabolites. **(A)** Flavonoid biosynthesis structural genes and metabolites network. **(B)** Signaling-related genes and metabolites network. **(C)** Transport related genes and metabolites network. **(D)** Hormone-related genes and metabolites network. **(E)** Transcription factor related genes and metabolites network. The network was visualized with the Cytoscape software (version 2.8.2).

To gain insight into the regulatory network of flavonoids and anthocyanins biosynthesis in purple and green leaves in tea plant, a correlation analysis was carried out between the concentration changes in the differential metabolites and the expression level changes of DEGs. For this analysis, DEGs annotated as involved in flavonoid metabolism, transcription regulation, transport, and hormone response, and differential metabolites including naringin, procyanidin, epigallocatechin gallate (EGCG), epigallocatechin (EGC), and derivatives of kaempferol, quercetin, and cyanidin, were selected (Figure 6 and Supplementary Table 4). The upregulation of the genes encoding MYB23 and bHLH96 in the purple leaves were highly correlated with the accumulation of flavonoids, including kaempferol 3-(2″-rhamnosyl-6″-acetylgalactoside) 7-rhamnoside, kaempferol 3-*O*-rutinoside, and quercetin 3-glucoside 7-xyloside. Two NAC transcription factors genes, *NAC008* and *NAC090*, were strongly correlated to procyanidin B3, kaempferol 3-*O*-rutinoside, quercetin, quercetin 4′,7-diglucoside, respectively, and EGCG. The expression of DEGs implicated in the hormone response, such as ethylene-responsive transcription factor (ERF), were closely correlated to the concentration of EGCG, quercetin, flavanone, kaempferol derivatives, myricetin 3,3′-digalactoside, and procyanidin B3 (Supplementary Table 4). The expression levels of five DEGs annotated as ABC transporter I family members were strongly correlated to the concentration of quercetin, EGCG, procyanidin B3, myricetin 3,3′-digalactoside, kaempferol 7-sophoroside, kaempferol 7-galactoside 3-rutinoside, quercetin 3-glucoside 7-xyloside, and quercetin 4′,7-diglucoside (Figure 6).

## Discussion

‘Zijuan’ is a special tea plant cultivar that produces purple-colored new shoots and green-colored mature leaves. The concentration of total anthocyanin in the purple leaves was significantly greater than in the green leaves (Figure 1B). This result is the same with the previous studies which suggest that the color difference between the purple leaves and the green leaves correlated mainly with anthocyanin accumulation (Li et al., 2017). Three major pigment classes, including chlorophylls, carotenoids, and flavonoids contribute to the color of the plant (Iwashina, 2015). A decreased concentration in the flavonoids and anthocyanins causes the leaf color change from purple to green. The purple tea plant leaves were found to have a significantly higher concentration of total phenolic compounds, flavonoids, and anthocyanins, whereas the green leaves were found to have a higher concentration of porphyrin, chlorophyll, and carotenoids (Kerio et al., 2013; Kilel et al., 2013; Shen et al., 2018). In our study, the compositions of the flavonoids downstream including quercetin 3-glucoside 7-xyloside, quercetin 7-glucuronide 3-rhamnoside, quercetin 3-(4″-acetylrhamnoside) 7-rhamnoside, kaempferol 3-gentiobioside 7-rhamnoside, kaempferol 3-(2″-rhamnosyl-6″-acetylgalactoside) 7-rhamnoside, and kaempferol 7-galactoside 3-rutinoside were quite different between the purple and green leaves, suggesting that these metabolites likely play an important role in determining the leaf color in ‘Zijuan.’ Some of the flavonoids and anthocyanins in plants are responsible for the purple, red and blue colors; when their contents are very high, they cover the green color of the chlorophylls (Iwashina, 2015). The purple flower color may be caused by copigmentation with flavonol glycosides such as quercetin and kaempferol (Williams et al., 2002). In our study, several kaempferol and quercetin derivatives including kaempferol 7-galactoside 3-rutinoside, kaempferol 3-gentiobioside 7-rhamnoside, kaempferol 3-(2″-rhamnosyl-6″-acetylgalactoside) 7-rhamnoside, quercetin 7-glucuronide 3-rhamnoside, quercetin 3-glucoside 7-xyloside, and quercetin 3-(4″-acetylrhamnoside) 7-rhamnoside were more abundant in the purple leaves than in the green leaves. The purple leaves of ‘Zijuan’ might be expressed by copigmentation between kaempferol and quercetin derivatives.

Flavonoids are biosynthesized from the phenylpropanoid pathway. The higher expression level of *PAL* and *4CL* in the purple leaves resulted the production of p-coumaroyl-CoA and provided adequate precursor metabolites for flavonoid biosynthesis in the purple leaves (Figure 3 and Supplementary Table 3), which might be the key reasons for the higher concentrations of anthocyanin, flavonol and flavone in the purple leaves. Chalcone isomerase (CHI) catalyzes the transformation of chalcone to naringenin. The higher expression level of *CHI* in the purple leaves could biosynthesize a large amount of naringenin in the purple leaves and provided the intermediates for the downstream biosynthesis of flavones, flavonols, and anthocyanins. F3H, F3′5′H, and F3′H are three vital enzymes in the flavonoid biosynthesis pathway, catalyzing the formation of hydroxylated derivatives such as dihydroquercetin and dihydromyricetin (Wang et al., 2014). A high expression level of *F3H* in the purple leaves facilitated the biosynthesis of flavones, flavonols, and anthocyanins (Figures 3A,B). All the DEGs which encoding F3H were expressed at a higher level in the purple leaves than in the green leaves. Compared to the green leaves, the purple leaves had a higher concentration of procyanidin B3, quercetin derivatives, including quercetin 7-glucuronide 3-Rham, quercetin 3-glucoside 7-xyloside, and quercetin 3-(4″-acetylrhamnoside) 7-rhamnoside, and kaempferol derivatives, including kaempferol 7-galactoside 3-rutinoside and kaempferol 3-(2″-rhamnosyl-6″-acetylgalactoside) 7-rhamnoside (Figures 2C, 3A), indicating that F3H was the main hydroxylase in the tea plant and plays key roles in flavonoid biosynthesis by regulating the flux through procyanidin and quercetin. FLS catalyzes the transformation of dihydroquercetin and dihydrokaempferol into quercetin and kaempferol, respectively (Tian et al., 2015). The transcription levels of *FLS* in the purple leaves were significantly higher than those in the green leaves, and the concentration of quercetin derivatives in the purple leaves, such as quercetin 7-glucuronide 3-rhamnoside, quercetin 3-glucoside 7-xyloside, and quercetin 3-(4″-acetylrhamnoside) 7-rhamnoside were significantly higher than that in the green leaves (Figures 2C, 3A), suggesting that FLS might play key roles in production of quercetin by dihydroquercetin in the tea plant.

Transcription factors, including GATA, bHLH48, NAC008, MYB, and WRKY, which were strongly correlated with differential metabolites, were demonstrated to be strongly upregulated in the purple leaves compared with the green leaves (Supplementary Table 4). Studies on the regulation of flavonoid biosynthesis in diverse plant species have demonstrated that MYB/bHLH complexes are involved in the regulation of flavonoid biosynthesis in a conservative manner across species (Nesi et al., 2001). Researchers have found that a WD40-repeat protein, a R2R3-MYB transcription factor and a bHLH-type regulation factor can form a WD40/R2R3-MYB/bHLH complex, which is involved in the control of flavonoid pathways in different plants (Jaakola, 2013). R2R3-MYB transcription factors in the tea plant are take part in flavonoid biosynthesis by activating the expression of flavonoid-correlated structural genes (He et al., 2018; Wang et al., 2018). In this study, the upregulation of the genes encoding MYB23 and bHLH96 in the purple leaves highly correlated with the accumulation of epigallocatechin gallate, quercetin 3-glucoside 7-xyloside, procyanidin B3, quercetin 3′-methyl ether, and quercetin 4′,7-diglucoside, which might be regulated by the WD40/bHLH/R2R3-MYB-mediated pathway. The NAC transcription factor can induce the transcription of the R2R3-MYB gene and lead to anthocyanin accumulation in the peach (Zhou et al., 2015). In Arabidopsis under high-light stress, researchers have found that the NAC transcription factor was involved in the induction of genes correlated to flavonoid biosynthesis, resulting in anthocyanin accumulation (Morishita et al., 2009). In our research, we found two NAC transcription factors gene *NAC008* was strongly correlated to EGCG and quercetin 3-glucoside 7-xyloside, respectively. This might indicate that the NAC transcription factor could also regulate flavonoid biosynthesis in the tea plant.

During fruit ripening, the color changes is accompanied by anthocyanin accumulation and rapid ethylene production (Faragher and Brohier, 1984). Previous research has showed that exogenous treatment with an ethylene-releasing compound could enhance the expression of the flavonoid biosynthesis pathway genes such as *CHS*, *F3H*, *ANS*, and *UDPG flavonoid glucosyl transferase* (*UFGT*) and the anthocyanin was accumulated in grape skins (He and Giusti, 2010). Among the DEGs correlated to the flavonoid biosynthesis pathway, homologs for *CHS*, *F3′5′H*, *FLS*, and *LAR* were more strongly upregulated in the purple leaves than in the green leaves (Figure 3B). The concentration of total polyphenol, flavonoids, and anthocyanins were higher after treatment with ethylene precursor 1-aminocyclopropane-1-carboxylicacid (ACC) (Ke et al., 2018). These results indicated that ethylene might promote the biosynthesis of flavonoids in the tea plant leaves.

Flavonoids are transported into the vacuole after synthesized in the cytosol (Zhao, 2015). To avoid toxicity, flavonoids, which are biosynthesized in the cytoplasm, are transported into vacuoles *via* transporters for isolation or storage. In plants, the ATP-binding cassette (ABC) transporter protein is one of the most important class of transporters. ZmMRP3 (multidrug resistance-associated protein 3), which is an ABC-type transporter protein, has been identified to be taken part in the flavonoids transport in *Zea mays* (Goodman et al., 2004). The expression levels of DEGs annotated as ABC transporter I family members were strongly correlated to the concentration of epigallocatechin gallate, flavanone, kaempferol 3-(2″-rhamnosylgalactoside) 7-rhamnoside, kaempferol 3-(2″-rhamnosyl-6″-acetylgalactoside) 7-rhamnoside, and these transporter genes might take part in the transmembrane transport of anthocyanins and flavonoids in the tea plant (Figure 6).

In conclusion, this study performed an integrated metabolome and transcriptome analysis of the purple leaves and the green leaves of the ‘Zijuan’ tea plant. Due to the higher expression levels of genes involved in the phenylpropanoid and flavonoid biosynthesis pathways in the purple leaves, there were significantly higher concentrations of flavonoids (especially kaempferol and quercetin derivatives) in the purple leaves compared with the green leaves. By correlation analysis, DEGs, which showed expression levels that strongly correlated with the concentrations of flavonoid, were identified, including transporters and transcription factors such as *NAC008*, *MYB23*, *bHLH96*, and *ABC transporter I*, which might be involved in flavonoid biosynthesis or transport. In conclusion, this study provides a new insight into the mechanism of the biosynthesis and accumulation of flavonoids and anthocyanins in the tea plant, and also constructed a correlation network of transcriptional expression levels and metabolite concentrations which could be used to apply genetic approaches to clarify the mechanism of flavonoids and anthocyanins regulation.

## Data Availability Statement

The datasets presented in this study can be found in online repositories. The names of the repository/repositories and accession number(s) can be found below: National Center for Biotechnology Information (NCBI) BioProject database under accession number PRJNA661139.

## Author Contributions

CL and HL designed the experiments and coordinated the project. SS and YT collected all the samples and performed the transcriptomic and metabolomic analyses. LG and DT performed the data analysis and qRT-PCR experiments. JL and YW performed the anthocyanin analysis. CL and FG wrote and edited the manuscript. All authors have read and approved the final manuscript.

## Conflict of Interest

The authors declare that the research was conducted in the absence of any commercial or financial relationships that could be construed as a potential conflict of interest.

## Publisher’s Note

All claims expressed in this article are solely those of the authors and do not necessarily represent those of their affiliated organizations, or those of the publisher, the editors and the reviewers. Any product that may be evaluated in this article, or claim that may be made by its manufacturer, is not guaranteed or endorsed by the publisher.

## Footnotes

1
http://metlin.scripps.edu

2
https://metlin.scripps.edu/
